# The Importance of Males to Bumble Bee (*Bombus* Species) Nest Development and Colony Viability

**DOI:** 10.3390/insects11080506

**Published:** 2020-08-05

**Authors:** Joseph E. Belsky, Allison A. Camp, David M. Lehmann

**Affiliations:** 1 Public Health & Environmental Systems Division, Integrated Health Assessment Branch Center for Public Health and Environmental Assessment (CPHEA), US—Environmental Protection Agency, Research Triangle Park, Durham, NC 27711, USA; belsky.joseph@epa.gov; 2 ORISE Researcher, Research Triangle Park Oak Ridge Associated Universities, Research Triangle Park, Durham, NC 27711, USA; aacamp@ncsu.edu

**Keywords:** *Bombus*, bumble bee, male, mating, immunity, queen success

## Abstract

**Simple Summary:**

Populations of some bumble bee species have declined over the last decade. Recognizing the importance of bumble bees to agriculture and natural ecosystems, there has been an upwelling of research to better understand the underlying reasons for observed population declines. While most research has addressed the health of bumble bee females (i.e., workers and queens), males have been largely ignored. Here, we explore the available published literature on the role males play in improving queen health and reproductive fitness, as well as in overall nest success. We conclude that males serve a unique and important role in bumble bee colony success.

**Abstract:**

Bumble bee population declines over the last decade have stimulated strong interest in determining causative factors and necessary conservation measures. Research attention has largely been directed toward bumble bee worker and queen health and their contributions to population stability, while male bees (i.e., drones) have typically been overlooked regarding their role in influencing colony fitness and longevity. In this review we assess existing literature on the diverse role of males within bumble bee nests and their importance to queen health and fitness, as well as to overall nest success. The implications of reproductive measures, including sperm transfer, mating behavior, mating plugs, and male immunity, among other topics, are examined. Overall, bumble bee males are found to drive colony function in a unique manner. Current knowledge gaps pertaining to the role of males are discussed. We highlight the importance of drones to queen success and fitness in many ways, and suggest future research exploring impacts of this often-neglected caste.

## 1. Introduction

Bumble bees have been increasingly used for crop pollination services in recent years. Many studies demonstrate largescale benefits in pollen transfer and ultimately fruit yield as a result of supplementing crop pollination with key species of commercially managed bumble bees [1,2,3]. In North America, the common eastern bumble bee (*Bombus impatiens*) is the commercially available bumble bee species used to pollinate blueberry, pumpkin, and sweet pepper crops [4,5,6]. Similarly, wild bumble bees provide valuable pollination services to crops [7,8] and to native ecosystems [9,10].

Declines in wild bumble bee populations have raised concern among biologists, prompting investigation into the factors contributing to the observed decreases. Recent additions of eight species of wild bees, including the rusty patched bumble bee (*B. affinis*), to the US endangered species list, and the imperiled status of franklin’s bumble bee (*B. franklini*) on the International Union for Conservation of Nature’s red list of endangered species, highlight the severity of these declines [11]. Stressors including pesticides, pathogens, habitat destruction, and nutritional deficiencies have been implicated [12,13,14]. Within the existing bee literature, drones are often overlooked and not included in research studies, with the thought that the caste is only important for copulation and little more. However, given the life cycle of bumble bees, drones likely play a more critical role than their research attention currently suggests.

There are more than 250 species of bumble bees worldwide and there is substantial variation in reproductive, developmental, behavioral, and ecological traits between species [15]. Consequently, we can only provide a basic overview of the bumble bee colony lifecycle. Most bumble bees have an annual lifecycle which starts with a fertilized queen exiting diapause during the late winter or early spring. After emergence and while searching for a nest site, the queen consumes pollen and nectar to replenish her depleted fat reserves and to promote development of her ovaries [16]. Once located, the queen provisions the nest with a ball of pollen mixed with nectar and builds a single nectar pot composed of wax. Within close proximity to the nectar pot, the queen will lay her first clutch of eggs, typically 6–16, in the ball of pollen and then incubate them with her body heat. Over the course of 3–5 weeks, the eggs will develop into larvae and later pupate before emerging as her first worker offspring. Newly eclosed workers assume foraging and brood rearing responsibilities, enabling the queen to concentrate on egg laying. Early spring bumble bee colonies are comprised of a single fertilized queen, female workers, and immature brood [17]. Within the nest, pollen is stored in clumps upon which brood are reared, and nectar pots are constructed to store nectar and wax [17]. Depending on the species, nests may grow large (i.e., 300–400 workers) or may peak with a population of approximately 100 workers [16]. During late summer to early autumn, the colony switches to producing “reproductives” (i.e., males and gynes). Male offspring are produced first followed by gynes (i.e., unfertilized queens). Gynes mate with males from other colonies and, once fertilized, these new queens enter diapause for the winter. Old queens, workers, and males die off at the end of the season, leaving only the newly mated gynes to act as foundresses in the spring.

The importance of males to the seasonal lifecycle of bumble bees may seem minor, but their role in colony success is underappreciated. Further, since honey bee biology and lifecycle are distinct from bumble bees, it is difficult to extrapolate what is known about honey bee drones to bumble bees. For instance, honey bee males only mate once, while bumble bee males have been documented to mate multiple times. Additionally, honey bee queens mate with between 10–14 males, while bumble bee queens can be either monandrous or polyandrous [18,19,20]. In terms of life cycle, honey bees live in large perennial colonies containing thousands of workers where a healthy fertilized queen can live 2–5 years [20]. Conversely, as outlined above, bumble bee colonies are annual and newly emerged gynes must mate in the fall. Young mated queens are the only caste to overwinter [18,19] and bumble bee colonies are not capable of replacing their queen when necessary.

**Figure 1:**
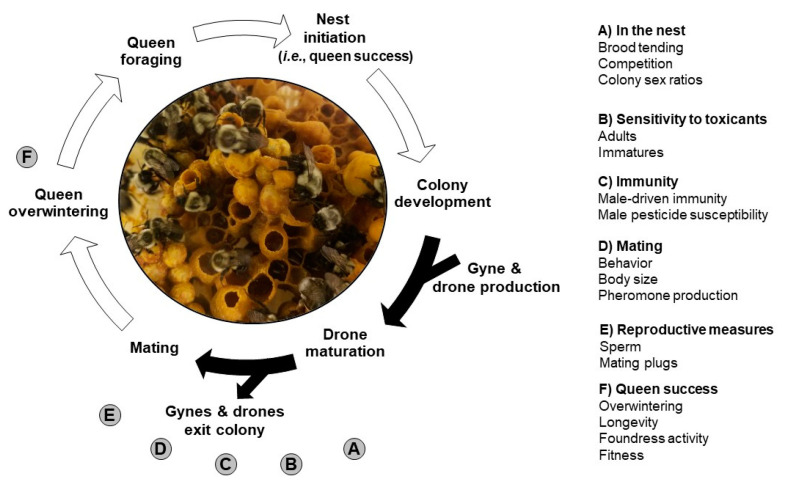
Bumble bee lifecycle and drone contributions to nest success. Bumble bee queens, the only caste to overwinter, emerge in late winter/early spring to begin foraging in order to initiate nests. Arrows indicate the progression of annual nest growth and development. Filled arrows indicate the presence of drones within the nest. Open arrows indicate events that are fulfilled by the queen. Aspects of nest growth, development, and success where drones make important are indicated by letters.

In this review, we discuss the scientific literature investigating bumble bee males using the lifecycle presented in Figure 1 as a guiding framework. Specifically, we explore the various contributions that males make within a colony including their role within the nest, their behavior, reproductive measures, and immunity, and their contribution to queen success. Knowledge gaps that are not addressed in the current literature and suggestions for future research are discussed. Further, we address risk assessment considerations, as well as the disparate data available for *B. terrestris* versus *B. impatiens* to clarify research needs for these two commercially important species. Overall, our review emphasizes the need for future studies examining links between males and colony fitness and viability.

## 2. Methods

We used Google Scholar, Web of Science, and ProQuest Agricultural and Environmental Science Database to find peer-reviewed publications through January 2020. The search strings (‘bombus males’ or ‘bumble bee males’ or ‘bumblebee males’ or ‘bombus drones’ or ‘bumble bee drones’ or ‘bumblebee drones’) and (‘semen’ or ‘sperm’ or ‘haplo-diploidy’) and (‘sex-ratios’ or ‘queen-worker conflict’) and (‘pesticides’ or ‘toxicology’) and (‘immunity’ or ‘pathogen’) were used. Of the 223 references that were identified, duplicates, conference abstracts and publications that were not relevant (i.e., did not contain bumble bee drone-specific data) were excluded. Peer-reviewed publications were prioritized; however, one conference document was included in this review [21]. The resulting list of publications included 111 citations. An additional 13 publications providing data from bumble bee microcolony [14,22,23,24,25,26,27,28,29], queenright colony [30,31] studies and relevant testing guidelines [32,33] were found by reviewing the reference section of [34]. Four publications providing background on honey bee males were also included in this review [20,35,36,37]. Publications discussed in this review are specifically focused on male bumble bee biology, their role within the nest, sensitivity to toxicants, immunity, mating behavior, reproductive measures, and their combined impact on queen success and colony viability (Table 1).

## 3. Drone Biology

Bumble bees, like other eusocial species in the order Hymenoptera have haplodiploidy sex determination where males result from haploid unfertilized eggs and females (queens and workers) result from diploid fertilized eggs [16]. Bumble bees begin to produce males at the end of the summer or the beginning of the fall [9]. Drone development time from egg to adult bee varies between species but requires, on average, approximately 24–28 days [15,38,39]. Within a species, development may vary due to brood nest temperature, number of attending workers, and the quality of available nutritional resources [9]. The density of workers in the brood area is thought to trigger queens to lay haploid (male) eggs [40]; conversely, male pupal emergence is proposed to trigger the rearing of queen larvae [41]. When drones eclose, they are still sexually immature as they must transfer sperm to their accessory testes; full sexual maturation occurs over 6–20 days [42,43,44]. Sexually mature drones leave their natal nest to mate with queens from other nests and do not return. Lifespans of bumble bee drones vary depending on individual species and seasonal fluctuations in different climate regions [38,39].

## 4. Role within the Nest

Bumble bee males contribute to nest function and dynamics within their natal nest (Figure 1A). When drones eclose from the brood mass, there is evidence that these young males actively participate in caring for immature brood. Researchers have found that *B. griseocollis* males participated in pupal incubation during their first few days post-eclosion, behaving similarly to workers performing the same task [45]. Male incubation increased pupal temperature by 4–6 °C. Although males were incapable of warming pupae to the extent of workers and queens, their participation was crucial to pupal warming. While this task may seem relatively trivial, brood incubation is essential for maintaining optimal brood growth rates. Within bumble bee nests, suboptimal temperatures for brood can disproportionately impact pupal development, and while cooler temperatures may not impact pupal survival, development time will be altered [46]. Taken together, the additional incubation help from drones may ensure that brood, potentially including gyne brood, are maintained at an optimal temperature.

As a bumble bee nest transitions during the season from producing primarily worker offspring to reproductives, the sex ratios of the nest begin to skew concordantly [16]. Since both workers and queens can lay haploid eggs that will develop into drones, the commencement of male production results in intra-nest conflicts that have implications for colony dynamics. While competition for male egg-laying occurs between workers and the queen, studies in *B. hypnorum*, *B. melanopygus*, and *B. terrestris* have all found that queens are ultimately dominant and are responsible for most male production within a nest [47,48,49,50]. Researchers have examined this shift to male production and have found evidence that when *B. terricola* and *B. melanopygus* have weak colonies that lose their queen early in the season, sex ratios will bias toward males far earlier than queenright colonies [51].

Another factor affecting the sex ratios within bumble bee nests is protandry (males are produced earlier in the season than gynes). This phenomenon has been observed for many bumble bee species [52]. Models postulated by Bulmer have suggested that the emergence of protandry may be under sexual selection pressures since males that emerge early will be available to mate with gynes as soon as they emerge [53,54]. Additional research investigated the male-biased sex ratios in bumble bees and concluded that it is likely that the male-bias is in part due to alterations in energy allocations within different nests that sometimes favor male production. Further, it is likely that both protandrous and protogynous (females produced before males) colonies coexist ensuring a form of biological balancing within the greater population [55]. Taken together, the appearance of males within a bumble bee nest relies on multiple factors and can be impacted by colony status.

## 5. Sensitivity to Toxicants

Routes of pesticide exposure for bumble bee drones are presumed to be the same as for workers [15], however, given the differences in caste development duration, adult size, and activity levels, attempting to apply worker food consumption rates to drones seems imprudent. Bumble bee drones may be exposed to pesticides developmentally and as adults, both within the natal nest and when foraging after leaving the natal nest. Pesticides can enter the nectar and/or pollen of plants following direct application, dust deposition on flowers, or through translocation from pesticide-treated seeds [Discussed in 15]. Foragers bring these pesticide-contaminated nutritional resources back to the colony where they are fed to developing brood and consumed by workers and presumably by eclosed drones that have yet to leave the natal nest. After leaving the natal nest in search of a mate, drones are known to forage [56,57,58] where they may be exposed to pesticides by direct contact or inhalation of spray, dust particles, or volatilized residues from plant surfaces [15]. The pesticide dose received by an individual drone will depend on the type and quantity of food consumed. Although there are data available for daily food consumption rates for *Bombus* worker larva and adults, we are not aware of any studies documenting food consumption rates for *Bombus* drone larva or adults [15]. 

### 5.1. Sensitivity of Mature Drones to Pesticides

Acute and sub-lethal toxicity of pesticides to bumble bee workers has been documented in several studies over the last decade [22,23,59,60]. However, little work has been conducted to examine direct pesticide toxicity to adult bumble bee drones (Figure 1B). One study directly tested *B. impatiens* drone sensitivity to the neonicotinoid clothianidin, and found survival was reduced at the daily field-realistic rate of 4.0 ng/g per bee [61]. Other studies have documented reductions in drones in experiments examining the effects of pesticides on colony structure of queenright colonies. For instance, reductions in the proportion of drones has been found after exposure to neonicotinoids [62,63,64]. Overall, additional work is needed to understand the effects of toxicants on adult drones, including both lethal and sub-lethal effects.

### 5.2. Sensitivity of Immature Drones to Pesticides

Immature life stages of bees, including egg, larva, and pupa, are also sensitive to pesticide exposures (Figure 1B). As outlined previously, developing males will be exposed to contaminants present in nectar and pollen collected by the foraging workers. Despite intensively searching the available literature, only one field study investigating the effects of pesticide exposure on drone production was found. *Bombus terrestris* drone production was affected both positively and negatively by thiamethoxam in a country specific manner [65]. Many studies have examined the effects of pesticide exposure on developing drones via the microcolony model. When isolated from their queen, small groups of bumble bee workers form a “microcolony” and lay unfertilized eggs that produce viable drones [34]. Many studies using this methodology have evaluated brood development. For instance, reduced brood production (reduced numbers of eggs and larvae) has been found to occur with exposure to neonicotinoids [24,66], and chitin synthesis inhibitors [67,68,69]. Another measurement used in microcolony studies, drone production, integrates all aspects of drone growth and development from egg to emergence. Some researchers choose to assess drone production rather than brood development since total offspring production is somewhat easier to measure given that bumble bee nest structures are layered, and quantifying brood structures can be challenging. Chemicals found to inhibit brood production also reduce overall drone production such as chitin synthesis inhibitors [69] and neonicotinoids [21]. Other chemicals found to reduce drone production are transgenic plant proteins [25], the ryanoid insecticide chlorantraniliprole [26], acaricides [70], biopesticides [71], the insecticides spinosad and spinetoram [27], and the pyrethroid λ-cyhalothrin [21]. And finally, drone body weight may be used as a measure of pesticide effects. Studies with the insecticides azadirachtin [28] and λ-cyhalothrin [29] have found that exposure to these chemicals reduced drone body weight. Reductions in body weight may have implications for male mating success, depending on the species. While much valuable data regarding the sensitivity of immature drones to pesticides has been gathered from microcolony studies, the degree to which microcolony studies recapitulate full colony exposures is unclear [34]. However, in the absence of full colony data, the microcolony data demonstrate that drones are sensitive to chemical insults and that drone outcomes can be compromised as a result.

## 6. Immunity

Bumble bees are exposed to an array of pathogens in their environment (Figure 1C). They defend themselves against these disease-causing pathogens through the action of an innate immune system that is composed of humoral and cellular branches [72]. Cellular immunity involves actions such as hemocyte-mediated phagocytosis, nodulation, and encapsulation of pathogens [73]. In contrast to cellular immunity, humoral immunity involves synthesis and secretion of immune mediators into the body fluid (i.e., hemolymph). Humoral immune defense mechanisms include melanization of hemolymph, mediated by phenoloxidase activity, and synthesis and secretion of antimicrobial peptides (AMPs) [74].

Like honey bees, bumble bees have far fewer canonical immune genes relative to solitary insects [75]. Consistent with Bateman’s principle of greater investment in female immunity, many immune genes are expressed more strongly in queens than males [75]. Irrespective, bumble bee male immunity is important for reproductive success and has been found to differ from workers. Researchers have determined that male encapsulation responses were lower than workers and this may mean that males are more susceptible to immune threats and parasitism [76]. However, male *B. terrestris* offspring originating from immune-challenged colonies had increased phenoloxidase activity but comparable antibacterial activity and hemocyte counts compared to controls [77]. Therefore, immune-challenged natal colonies appear to produce adult males with somewhat enhanced immunity.

The energetic trade-off between immune function and reproductive function in bumble bee drones has been investigated. Wilfert and colleagues examined whether sperm quantity and immune function were subject to an energetic tradeoff within *B. terrestris* [78]. Interestingly, they found a positive correlation between sperm production quantity and antibacterial activity against a pathogen, suggesting that there is not a trade-off between these measures and that males with high sperm quantity also have robust immune function.

There is evidence that pesticide exposure may also impact drone immune measures. Research conducted with *B. impatiens* exposed to the neonicotinoid pesticide clothianidin analyzed transcriptomic responses to the exposure using RNAseq [61]. Exposure to clothianidin altered expression of genes relating to a variety of biological functions, including immunity. While this work did not provide insights into the functional repercussions of these changes, it highlights an area lacking adequate data, and also provides evidence that there are important differences between workers and males and they must be studied independently [61].

## 7. Mating Behavior

Drone mating behavior and related factors are critical for successful queen fertilization and subsequent nest foundress activity by new queens (Figure 1D). After leaving their natal nest, drones of some species patrol an area several hundred meters from their nest leaving scent markings along the way [19,79] whereas other species (i.e., *B. hypnorum* and *B. muscorum*) congregate at nest entrances seeking to mate with gynes as they exit or enter the nest [19,80,81]. Mating success of male *B. terrestris* has been shown to be affected by ambient temperature, age, weight, and virginity [82]. For example, the highest mating percentage occurred at 23 °C, whereas the lowest mating percentage was observed at 29 °C [83]. Unlike honey bee drones, which can only mate once and perish thereafter, bumble bee drones can mate multiple times [84]. This ability has important impacts on genetic diversity. Male *B. terrestris* store enough sperm (i.e., 600,000) in their accessory testes to inseminate more than one queen [43]. The spermatheca of once-mated *B. terrestris* queens contain between 40,000 and 60,000 sperm [43]. Further, drone multiple mating has been found to be beneficial for *B. terrestris* colonies and researchers have found that queen mating with non-virgin drones increased queen colony foundation and fitness [85]. Some species of bumble bees also have the capability of detecting nestmates during mating in order to avoid inbreeding. Evidence suggests that *B. muscorum* [86], *B. frigidus*, and *B. bifarius* are able to avoid inbreeding, while *B. californicus* and *B. rufocinctus* may mate with nestmates [87]. The implied low evolutionary selection pressure to avoid nestmates during breeding has the potential to reduce genetic diversity within *B. californicus* and *B. rufocinctus*, however it is unclear how frequently this scenario occurs in natural settings.

Both physical and chemical attributes of drones also influence their mating success. Research conducted with *B. terrestris* revealed that heavier and younger males copulated more rapidly and for shorter durations as compared to older and lighter males [83]. However, under situations of more intense competition, age was no longer important for mating success, and fore and hind tibiae lengths were predictive of reproductive success, with drones with longer leg lengths being more successful [83]. Adequate food resources within a drone natal nest will influence body size and related morphologic measures. Nests with limited resources, specifically pollen, which is critical for larval development, will produce smaller offspring [30,31].

Beyond phenotypic attributes, pheromone production for mate attraction is also important for drones. In bumble bees, male pheromone production has been found to be age-dependent and studies with *B. terrestris* and *B. lucorum* found that both species reached peak pheromone levels by seven days post-eclosion [88]. After day seven, *B. terrestris* pheromone levels dropped, whereas levels in *B. lucorum* did not. This suggests that *B. terrestris* may have a narrower window during which to find a mate than *B. lucorum*. Further, differences in pheromone chemicals were identified between the species, providing evidence for a means by which gynes can locate their conspecifics [88]. A follow-up study found that underlying differences in *de novo* synthesis of pheromones in the labial glands of these species controlled age-related changes in pheromone production [89]. Common hormones in immature insects are proposed to contribute to the biochemical pathway for pheromone production [90]. Therefore, stressors that disrupt or alter drone hormone production may have the potential to negatively impact subsequent mating and ultimately queen success.

## 8. Reproductive Measures

Drone reproductive measures include sperm production and quality, as well as mating plug production and composition (Figure 1E). These factors can have dramatic impacts on bumble bee queen success and are underappreciated aspects of drone biology. For instance, in *B. terrestris*, sperm can influence survival of fertilized queens [91], as well as hibernation success, queen longevity, and fitness [92]. It is unknown what aspect of sperm influences these effects observed in queens, and whether other factors such as drone age or gyne age also play a role.

One factor that is known to vary among males and may contribute to the observations of variable queen success after mating is sperm length. Sperm length, which is relatively uniform within one male, varies widely between siblings within a nest, between males of the same species from different nests, as well as between species [93]. Within *B. terrestris*, sperm length is positively correlated to body size, thus providing a means by which queens could select for a sperm length via drone size [94]. This suggests that for some species, sperm length may be a trait that is under sexual selection.

At the end of copulation, bumble bee drones place a gelatinous material into the genital tract of queens, and this is referred to as a mating plug [95]. This substance originates in the accessory glands of drones, and prevents the sperm of other males from entering the queens genital tract, thereby preventing sperm competition [96]. The mating plug of the monandrous *B. terrestris* is effective for this purpose [97], however, in polyandrous species, mating plugs are only partially effective such as with *B. hypnorum* [98]. Examinations of mating plug composition in *B. terrestris* have revealed that the predominant chemicals within the plug were fatty acids and one cyclic peptide (cycloprolylproline) [99]. It was determined that the cyclic peptide was not required for physical plug formation, but rather may influence queen post-mating behavior and reduce her receptivity to other males. This phenomenon of accessory gland products repressing female receptivity has been demonstrated in other insects such as *Drosophila melanogaster* [100]. Therefore, this chemical may drive the monandry of *B. terrestris*. A subsequent study identified linoleic acid as a compound within mating plugs responsible for inhibiting further queen mating [97]. These studies highlight the capability of drones to influence queen mating behavior as well as colony paternity in certain species.

## 9. Queen Success as a Result of Mating

As a result of mating, bumble bee drones directly impact subsequent queen success in several ways (Figure 1F). First, it is important to note that many species of bumble bees are polyandrous, including *B. hypnorum, B. breviceps*, and *B. perplexus* [98,101,102,103]. *B. terrestris* is generally monandrous although polyandry can occur [104,105]. There is evidence that queen polyandry (i.e., access to sperm diversity) may contribute to colony success [106,107]. Artificially inseminated *B. terrestris* queens challenged with parasitism revealed that colonies resulting from queens inseminated with high sperm diversity were less impacted by parasites and had greater reproductive success [108]. Separately, artificial queen polyandry in *B. terrestris* resulted in decreased levels of *Crithidia bombi* infestation in worker offspring in resulting colonies [109]. While these studies with *B. terrestris* do not replicate natural conditions (i.e., *B. terrestris* is monandrous), they provide evidence that polyandry imparts benefits to queen fitness and results in more robust bumble bee nests.

Drone-specific factors can also directly impact queen survival and longevity. For instance, studies examining five different patrilines of *B. terrestris* found that patriline impacted queen survival, with some patrilines significantly decreasing queen survival. [91]. Additional work with *B. terrestris* has corroborated the finding that patriline influences queen life span, and it was discovered that this effect was specific to sperm transfer and independent of accessory gland material transfer (e.g., mating plug) or mate guarding behaviors [92]. Notably, *B. terrestris* queens artificially inseminated with a mixture of sperm versus sperm from a single male exhibited reduced overwintering survival, suggesting that monandry may be closely tied to increased survival for this species [92].

Two studies have examined the effect of mating on survival and longevity. Virgin queens have been found to be significantly more likely to survive compared to their non-virgin counterparts [110], and virgin queens have been found to have increased longevity [111]. However, the effect was minor, and it is unclear whether a small difference in longevity constitutes a biologically significant difference. Further, virgin queens, while they were more likely to survive, would be unable to establish a full-fledged nest, therefore the utility of this work primarily pertains to bumble bee rearing operations.

There is also evidence that mating may also influence the immune system of queens. Molecular evidence has suggested that mating prior to overwintering for *B. terrestris* resulted in elevated levels of antimicrobial peptides, and these peptide levels were maintained throughout overwintering [112]. Conversely, comparisons between virgin and mating queens have found that mating significantly increases the incidence of melanized spermatheca, which may indicate pathogen transfer during mating [111]. Taken together, mating may have both benefits and draw backs for immunity in queens.

Importantly, drones have also been shown to impact foundress activity and productivity of the resulting colonies (i.e., queen fitness). Drone patrilines, which have been implicated in influencing survival, also impact fitness, and patrilines associated with lowest mean queen survival were also linked to lowest queen fitness [91]. Mating with non-virgin drones has also been found to improve the nest initiation of *B. terrestris* queens post-diapause [85]. The queens mated with non-virgin males also produced more workers and drones in their respective colonies, and thus improved fitness [85].

## 10. Discussion and Knowledge Gaps

While there are data to support the importance of drones to bumble bee queen success and therefore bumble bee population stability, there are a variety of areas for which critical data are absent. The most critical data gaps are discussed here.

### 10.1. Species Focus

Most current literature detailing the various ways that bumble bee drones impact colony foundation and fitness are focused on the commercially managed species *B. terrestris* that is native to Europe, while only a few studies have addressed these impacts on the commercially managed species *B. impatiens* that is native to North America. Expanding our understanding of *B. impatiens* drones is important given the widescale usage of *B. impatiens* for agricultural pollination. Although *B. terrestris* and *B. impatiens* are useful investigational models, they are not representative of all bumble bee species [113]. Beyond these two commercially available and agriculturally important species, other species have become available commercially (i.e, *B. ignitus* [114,115,116,117], *B. atratus* [118,119], *B. huntii* (Biopest group)), and additional species are being evaluated for this purpose (e.g., *B. hypocrita* [116,117,120], *B. pyrosoma* [121,122], *B. picipes* [121,122], *B. breviceps* [101], and *B. vosnesenskii* [123]). While the commercial availability of additional species would facilitate much more drone research, there are still hundreds of other bumble bee species worldwide that are understudied due to their smaller populations, challenges associated with laboratory rearing, and/or perceived lesser importance. Male bee biology may be an important factor in the population stability of these of these wild bees and necessitates additional research resources.

### 10.2. Effects of Pesticides

Pesticide exposure has been implicated in bee population declines, however most of the work within this area has focused on worker outcomes. There are many areas of research for which data on drones is of critical importance and currently lacking. The first is the effect of pesticide exposure on drone reproductive measures. No studies have determined whether drones exposed to pesticides within their natal nest have reduced reproductive output or resulted in behavioral detriments that would impede mating. For instance, studies that examine sperm production, pheromone production, mate finding, and mating behavior after exposure to pesticides are all needed. Similar studies on adult drones that have been exposed to pesticides once they begin foraging outside of their natal nests are needed as well. Given the ease with which drone brood can be produced using the microcolony model, assessments of this kind are accessible to researchers.

If drone reproduction and behavior are impacted by pesticides, then the effects of exposed drones on queen outcomes would be the logical next step. As we have described here, drone-specific factors can influence queen overwintering, survival, nest initiation, and ultimately fitness. It is currently unknown whether drone exposure to pesticides will also affect these measures. Mating studies used to explore this endpoint could also be used to evaluate transgenerational effects of drone pesticide exposure to determine whether offspring are impacted. Relevant endpoints for next generation workers and drones include development times, body size, immune function, foraging, and behavior within the nest. Relevant outcomes for drones include sperm parameters and mating behavior.

### 10.3. Immune System

There are many aspects of bumble bee drone basic biology that remain understudied. For instance, little work has been done on drone immune function. First, a better understanding of the fundamental differences between worker and drone immune systems is needed. Subsequently, conclusive studies are needed to determine whether pathogen transfer occurs during mating for bumble bees. It is known that this can occur in honey bees, and spores of both *Nosema apis* and *N. ceranae* have been found in semen and infected drones can infect queens as a result of copulation [35,36]. Given that many species of bumble bees are polyandrous, if pathogen transfer does occur during mating, some species will be more susceptible to infection.

### 10.4. Nutritional Requirements

Another component of drone biology that is poorly understood is the nutritional requirements of drone larvae and adults. No studies have examined the amount of food required to successfully rear drones, however estimates exist for workers [15]. Understanding the nutritional needs of males would increase our understanding of toxicant exposure that drones may encounter during development within the nest or while foraging, and may clarify the relationship between food consumption, development time, and body size.

### 10.5. Risk Assessment Considerations

Assuming responses observed in honey bees are predictive of other bee species, regulators rely on the honey bee as a surrogate species for pesticide risk assessment. However, there are differences in life history and phenology between honey bees and bumble bees. Importantly, these species may also differ in their sensitivity to pesticides. Consequently, reliance on honey bees as the model organism for pesticide risk assessment is being questioned [15]. To address these concerns, there is an ongoing international effort to develop standardized bumble bee-specific acute and chronic toxicity tests. Following the testing paradigm established for honey bees, new tests for evaluating acute pesticide toxicity in adult worker (i.e., female) bumble bees have been established [32,33]. Potential adverse effects on drones are not considered when using these screening-level tests. Currently, there are no accepted methods for assessing chronic toxicity or colony-level effects in bumble bees of any caste. While *Apis* bee researchers are beginning to recognize the importance of drones to colony reproductive health [37], bumble bee drones contribute far more to colony success than the act of insemination. Bumble bee drones are dynamic partners in the colony, making contributions to the genetic composition of the colony, maintaining brood nest temperature and, in stark contrast to honey bee drones, *Bombus* drones contribute directly to queen overwintering survival and by extension foundress activity. For these reasons, drones warrant significantly more research attention.

### 10.6. Future Research

Contrary to the scant research performed on drones, drones can be acquired from commercial vendors and are also easy to produce in the laboratory [34,124]. As mentioned previously, when workers are isolated, small groups will form a microcolony and lay unfertilized eggs that develop into drones [34]. These drones could be readily evaluated for pesticide acute toxicity (contact and oral) using existing protocols for workers. Studies could be devised to assess pesticide effects on drone behavior (brood tending and foraging). The effects of chronic developmental pesticide exposure on drone development, immune system function and sperm quality could also be studied by provisioning microcolonies with pesticide spiked pollen [34,69]. Critically, pesticide-exposed drones could be used in mating studies to confirm drone reproductive fitness. Lab-mated queens could then be artificially overwintered, evaluated for their ability to survive diapause and ultimately for their ability to act as a foundress. Studies like these would help inform risk assessment activities directed at protecting the health and viability of bumble bee colonies.

Additional work is needed to determine the degree to which males contribute to colony success at different stages of development. Given the lack of research on bumble bee drones, it is impossible to make estimates of their contribution currently. However, this information would not only give insight into the relative importance of drone health, but also provide clues about the impact of observed sex ratios and the effects of sex ratios within a colony.

## 11. Conclusions

Throughout this review we have assessed the current literature on the various ways in which male bumble bees drive colony foundation and fitness, as well as the variables impacting their individual physiological function. Our review compiles a list of bumble bee drone studies and, as a result, highlights the often-overlooked ways in which drones drive colony function. Secondarily, we discuss literature assessing how compromised drone physiology drives downstream colony function by way of offspring output and their associated function. Moreover, we bring forth a list of current knowledge gaps related to bumble bee drones which offer promising areas for future research studies. Scientific projects investigating these knowledge gaps are timely given the previously mentioned large declines in bumble bee populations. We hope that bumble bee researchers use the data gaps highlighted here to guide future research efforts to fill the most critical areas that lack drone-specific work.

## Figures and Tables

**Table 1:** Number of publications identified for each endpoint.

Endpoint Evaluated	Number of Publications
Drone biology	10
Role within the nest	13
Sensitivity to toxicants	37
Immunity	8
Mating behavior	17
Reproductive measures	10
Queen success as a result of mating	16

## Disclaimer

This article has been reviewed by the U.S. Environmental Protection Agency and approved for publication. Approval does not signify that the contents necessarily reflect the views and policies of the Agency or of the US Federal Government, nor does the mention of trade names or commercial products constitute endorsement or recommendations for use of those products. The authors report no financial or other conflicts of interest. The authors alone are responsible for the content and writing of this article.
